# Socio-Geography of Human Mobility: A Study Using Longitudinal Mobile Phone Data

**DOI:** 10.1371/journal.pone.0039253

**Published:** 2012-06-28

**Authors:** Santi Phithakkitnukoon, Zbigniew Smoreda, Patrick Olivier

**Affiliations:** 1 Culture Lab, School of Computing Science, Newcastle University, United Kingdom; 2 Sociology and Economics of Networks and Services Department, Orange Labs, Issy-les-Moulineaux, France; University of Zaragoza, Spain

## Abstract

A relationship between people’s mobility and their social networks is presented based on an analysis of calling and mobility traces for one year of anonymized call detail records of over one million mobile phone users in Portugal. We find that about 80% of places visited are within just 20****km of their nearest (geographical) social ties’ locations. This figure rises to 90% at a ‘geo-social radius’ of 45****km. In terms of their travel scope, people are geographically closer to their weak ties than strong ties. Specifically, they are 15% more likely to be at some distance away from their weak ties than strong ties. The likelihood of being at some distance from social ties increases with the population density, and the rates of increase are higher for shorter geo-social radii. In addition, we find that area population density is indicative of geo-social radius where denser areas imply shorter radii. For example, in urban areas such as Lisbon and Porto, the geo-social radius is approximately 7****km and this increases to approximately 15****km for less densely populated areas such as Parades and Santa Maria da Feira.

## Introduction

Mobile phone data have several interesting features that we exploit in this work. Crucially, caller connections generally correspond to everyday personal networks and are representative of people’s most important social relations. It is unusual for us to call somebody we do not know, but in such cases (e.g. certain businesses and commercial services) this ‘noise’ can be handled by taking account of call reciprocity [1]. Although a mobile phone personal network is not completely representative of a face-to-face social network (i.e., there are people who we meet and talk to on a regular basis but have never called, e.g., colleagues, neighbors, etc.), it reflects people’s face-to-face social network better than, for example, online social network ‘friends’ and ‘followers’ on Twitter, FaceBook, MySpace, Google+, etc., for which people can have hundreds of contacts that they have never physically met. A mobile phone is also a personal device, associated with an individual, as opposed to a fixed phone which is shared. Thus, when we make a call to a mobile phone user, we are calling a specific person, not a household or an office. Furthermore, cellular communications are geo-located by the serving antenna’s geographical position; this affords us insight as to the spatial organization of individuals and networks as well as people’s mobility.

An increase in the availability of mobile phone usage datasets in recent years has led to a number of studies of human mobility [2]–[8], social structure [1], [9], [10], mobility and social events [11]–[13], as well as investigations of social networks and economics [14], [15], social networks and mobility [16], [17], and the geography of social networks [18], [19]. This study reflects this trend and aims to further extend our understanding of the relationship between human mobility and social networks. In particular, geographical and social aspects of travel scope, i.e., visited places, including the nature and extent of what can be inferred about people’s travel scope from the geographical span of their social network (and vice versa). An understanding of human mobility has many applications. Cloud computing, content-based delivery networks [20], and location-based recommender systems [21]–[23] can all benefit from quantitative and qualitative knowledge of users’ locations. Accurate models of human mobility are also an essential element of urban planning [24], understanding human migration patterns [25], and epidemiology [26].

## Methods

### Dataset

In this study, we used an anoymized dataset of over 1.3 million mobile phone users (1,318,905) in Portugal that provides both fine-grained longitudinal mobility traces and communication logs over one year between 2006 and 2007. The data accounted for approximately 13% of the population and was collected for billing purposes in all 308 municipalities by a European telecom operator. To safeguard personal privacy, individual phone numbers were anonymized by the operator before leaving their storage facilities, and were identified with a security ID (hash code). Each entry in the dataset has a Call Detail Record (CDR) comprising the voice call information: Timestamp, Caller’s ID, Callee’s ID, Call duration, Caller’s connected cell tower ID, and Callee’s connected cell tower ID. The dataset does not contain information relating to text messages (SMS) or data usage (Internet). The location of the user was recorded as the nearest connected cellular tower location when the user made or received a call. There are 6,509 cell towers, each on average serves an area of 14****km^2^, which reduces to 0.13****km^2^ in urban areas such as Lisbon and Porto.

**Figure 1:**
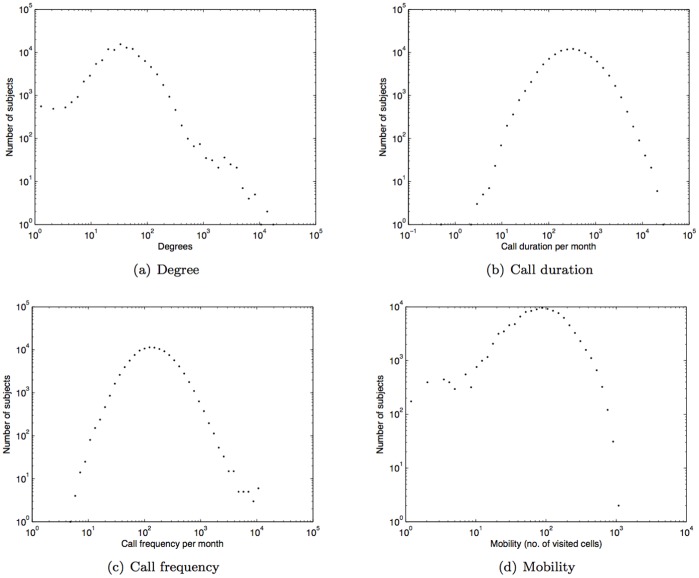
Histogram of degree, call duration (minutes), call frequency, and mobility.

To ensure that we used fine-grained mobility traces for subjects we selected only those mobile phone customers whose locations were recorded at least five times each month; this led to the consideration of 110,213 subjects from the dataset. Following [1] we only considered reciprocal communications in inferring the social network for each subject. On average, over one year, there are approximately 49 reciprocal links per subject; each subject spends 467 minutes on the phone each month (approximately 6 mins daily) and is connected in 173 calls monthly (approx. 8 calls daily) across 98 different cell towers. The histogram of degrees (i.e. number of ties), call duration per month (in minutes), call frequency per month, and mobility (i.e. number of different cells visited) are shown in Fig. 1(a), 1(b), 1(c), and 1(d), respectively.

### Geographical and Social Distance

Our principal concerns are the mobility and the social network of the subjects. Mobility relates to the nature and physical distance in of movement in geographical space, while the social network relates to the nature and number of tie strengths and companion relationships.

We first estimate the home location for each of the users as the cell tower location for which the user has the highest level of call activity during nighttime hours (10****PM thru 7****AM). An inherent limitation with our dataset is that the geographical location (latitude and longitude coordinates) of a connected cell tower is defined as the centroid of the cell tower’s coverage area. Furthermore, home location estimation is also influenced by a number of other factors, including the level to which the market share of our mobile phone operator reflects the general population, subjects’ call plans (which can influence number of calls at different time of the day), and the possibility of subject using multiple mobile phones.

**Figure 2:**
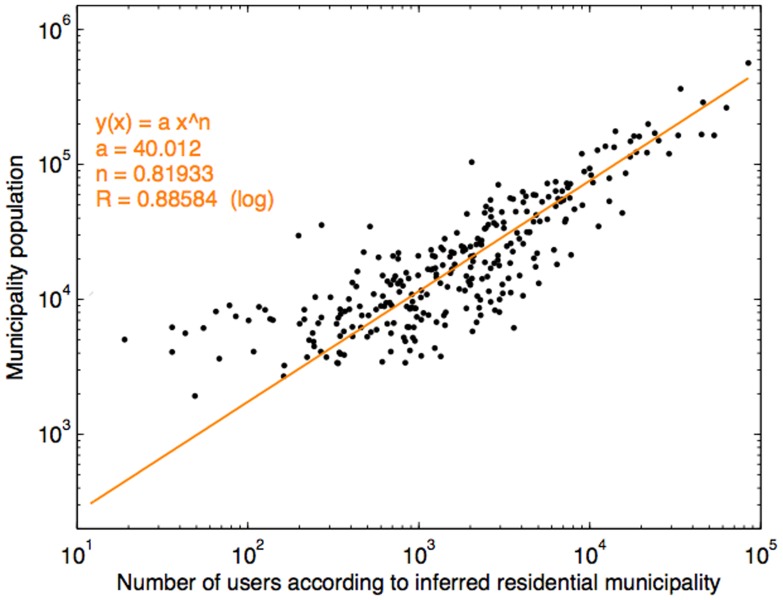
Comparison between our inferred residential municipality population of the mobile phone users and the actual municipality population obtained from census data [**27**].

Nonetheless, we attempted to validate the reliability of our home location estimation process by assigning all subjects to residential municipalities according to our estimation of their home locations and comparing the proportion of subjects assigned to different municipal areas with figures obtained from the Instituto Nacional de Estatística 2001 Census [27] (depicted in Fig. 2). The result shows, for the most part, a linear relationship with a few mismatches (which account for approximately less than 10% of the municipalities). Overall, this establishes the reliability of our approach to home location estimation for the subjects concerned.

**Figure 3:**
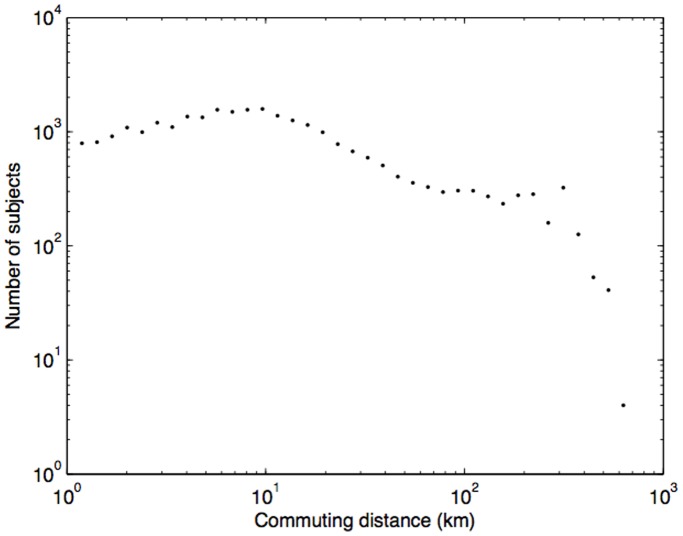
Histogram of commuting distance – distance between subjects’ home and work locations with the average of 7.59 km and 68% of the subjects have commuting distances of less than 10 km, which agrees with [**28**] **of ties.**

By applying a similar approach, we estimated a work location as the cell tower location with the highest call activities during normal business hours (between 9****AM and 5****PM). The efficacy of the work location estimation can in part be demonstrated by our calculation of *commuting distance* (distance between home and workplace), based on our home/work location estimation (shown in Fig. 3), for which 68% of the subjects having the commuting distance of less than 10****km corresponds with that reported in [28] (67.4% of the population in Portugal commute under 10****km).

**Figure 4:**
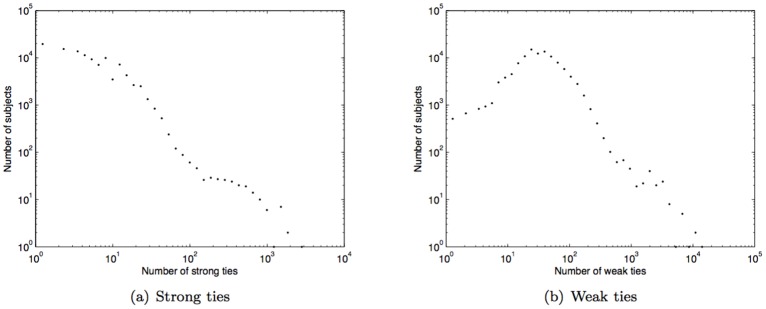
Histogram of strong and weak ties.

For social distance, we adopt the theory of tie strength developed by Mark Granovetter in his 1973 milestone paper [29]. Granovetter categorized ties into two types: *strong* and *weak*. Strong ties are people who are socially close to us and whose social circles tightly overlap with our own. Typically they are people we trust and with whom we share several common interests. By contrast, weak ties represent mere acquaintances. He defined the strength of a tie as “a combination of the amount of time, the emotional intensity, the intimacy (mutual confiding), and the reciprocal services” ([29], p. 1361). We adopted a similar approach to [1] using the amount of time spent in communication and reciprocity as proxies. By computing tie strength based on call duration normalized by the average as given by Eq. (1) we can categorize ties accordingly.
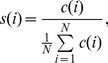
(1)where 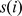 is the tie strength with tie *i*, 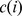 is the total call duration with tie *i*, and the denominator is the average call duration of all associated ties where *N* is the number of associated ties. We then classify ties whose 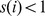 as *weak ties* while *strong ties* are ties with 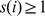. The histograms of strong and weak ties for our subjects are shown in Fig. 4(a) and 4(b); the average numbers of strong and weak ties are 7 and 42 respectively.

**Figure 5:**
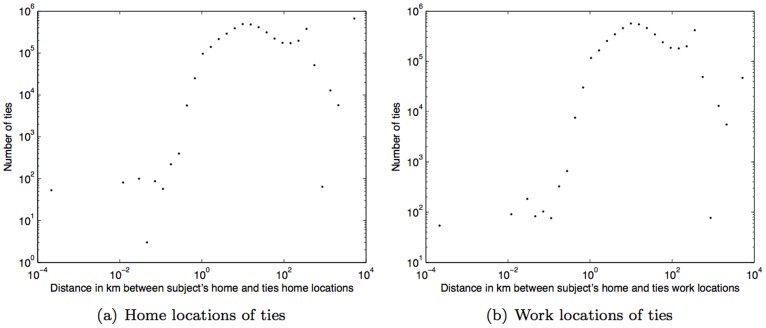
Histogram of geographical distance (km) between subject’s home location and home and work locations of ties.

The geographical distance between subjects and their social ties can be described by the histograms of the distances between their home locations and the home locations of their social ties (see Fig. 5(a), average distance of 19.66****km), and the distances between their home location and the work locations of their social tiles (see 5(b), average distance of 13.30****km). These distances are calculated in kilometers using the Haversine formula given by Eq. (2).
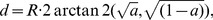
(2)where 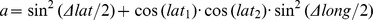, 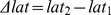, 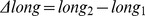, and *R* is the Earth’s radius (we used the average radius of 6,371****km).

**Figure 6:**
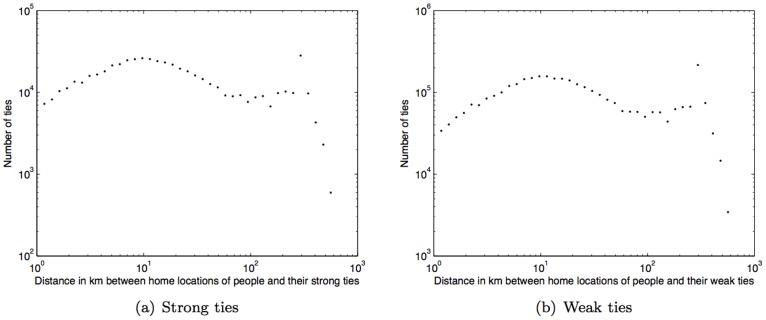
Histogram of geographical distance (km) between home locations of people and their ties.

Taking both geographical and social distances into account, we observe that on average our subjects live approximately 11****km closer to their strong ties than to their weak ties. Figures 6(a) and (b) show the histograms of distances between the subjects’ home locations and their strong ties’ and weak ties’ home locations, with average distances of 41.50****km (std. dev.  = 91.52****km) and 52.75****km (std. dev.  = 81.98****km), respectively.

## Results

**Figure 7:**
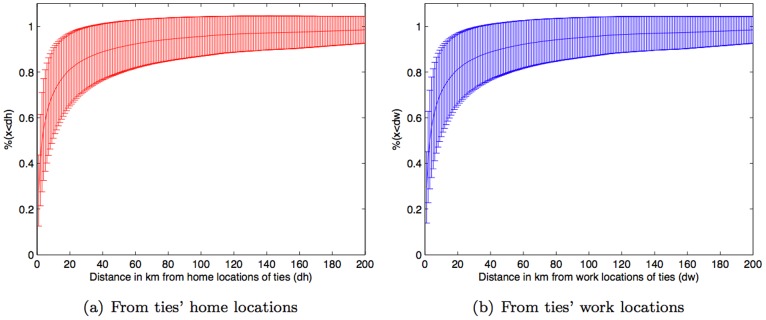
Percentage of travel scope being within some distance from ties’ locations, where distance varies from 0 to 200 km. Standard errors are shown in Fig. S5.

Although the location of a subject was collected only when the subject was connected to the cellular network, location traces from mobile phone data have been shown to be a reasonable proxy for individual human mobility [4]. The locations visited by people can be reasonably considered as their scope of travel and our primary interest is the geographical relation between a person’s *travel scope* and their social network. Following [1] we infer the social network for each subject based on reciprocal calls. We then consider the physical distance of these social ties with respect to travel scope. Home and work locations are reasonable proxies for the location of a tie as people normally spend most of their time at their home or workplace [30], [31]. Based on our home location estimations, Fig. 7(a) shows the average percentage of travel scope being within *dh* kilometers from a tie’s home location along with standard deviation bar, where we have computed this for values of *dh* between 0****km to 200****km. It turns out that *80% of travel scope is within just 20* **** *km of the nearest social ties’ home locations*. This rises to 90% at a *geo-social radius* of 45****km. A similar result also holds for the work locations of ties, as shown in Fig. 7(b). This suggests a strong relationship between people’s mobility and their social networks. In other words, *social network geography is indicative of an individual’s travel scope and vice versa*.

For a null hypothesis comparison, we constructed a null model in which subjects’ locations were randomly interchanged and it yielded relatively lower average values and much wider error bars (as shown in Fig. S1 along with the z-scores in Fig. S2).

**Figure 8:**
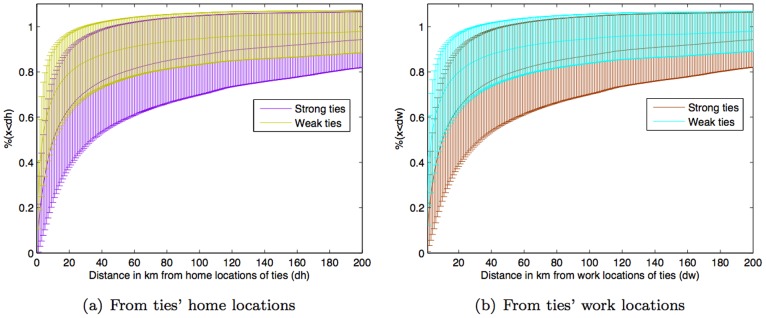
Percentage of travel scope being within some distance from weak and strong ties’ locations. Standard errors and p-values are shown in Fig. S6 and Fig. S10, respectively. Note: Fig. S7, S8, and S9 show standard errors for Fig. S1, S2, and S3, respectively.

Social tie strength also has an impact on the relationship between mobility and social network geography as *travel scope tends to be geographically closer to weak ties than strong ties*. This is the case for both home and work locations of ties as shown in Fig. 8. Specifically, the result shows that *people are approximately 15% (std. dev.  = 10.7%, p-value  = 0.09) more likely to be at some distance away from their weak ties than their strong ties*. Thus, although people tend to reside near their strong ties (Fig. 6), their mobility is biased towards the geographic locations of their weak ties. In addition, a supporting result based on *neighborhood ratio* (a portion of ties being in a certain vicinity of subjectÕs travel scope) is shown in Fig. S3. One may question about the result if it is influenced by the larger number of weak ties than strong ties per subject. A very similar result is also obtained by considering just only the strongest and weakest ties per subject, and shown in Fig. S4.

**Figure 9:**
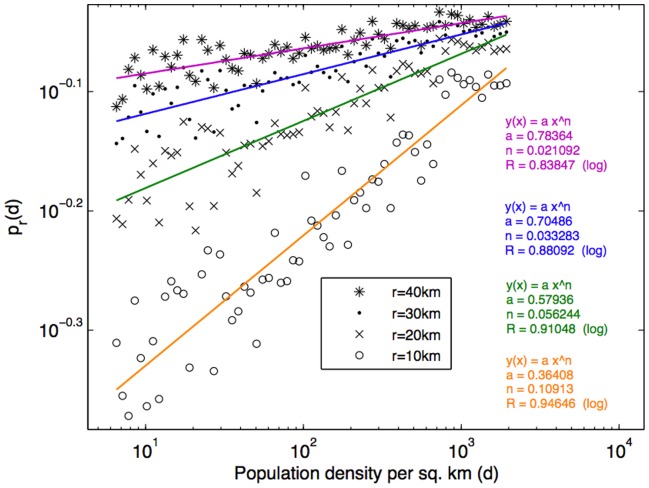
The likelihood of being within r km from a social tie 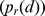 increases with the municipality population density (*d*), and this relationship can be fitted with the power law. At the same level of population density, the likelihood 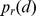 is lower for a smaller value of *r* (as shown with sample values of *r* from 10****km to 40****km). Meanwhile with a smaller value of *r*, the likelihood increases with the population density level at a higher rate.

Furthermore, it has been shown in [32] that areas with higher population density may provide greater trip-end concentrations, which minimize the necessity to travel outside the area, and may therefore reduce the travel scope. To further investigate the impact of the population density to the geo-social radius, for each of 308 municipalities we used population density statistics from the Instituto Nacional de Estatística 2001 Census [27] and considered the percentage of travel scope within a geo-social radius *r* across different population density levels, where *r* = 10****km, 20****km, 30****km, and 40****km. As Fig. 9 shows, the percentage (likelihood) increases with population density where the trend can be fitted with a power law distribution. It can also be observed that shorter geo-social radii have a lower likelihood in low population density areas. However, these shorter geo-social radii increase with the population density at faster growth rates – which is in line with the scaling phenomena in city growth described by Zipf’s law for cities [33]. Interestingly, *in densely populated areas (i.e., urban areas) 80% of travel scope is within a 10* **** *km geo-social radius*.

**Figure 10:**
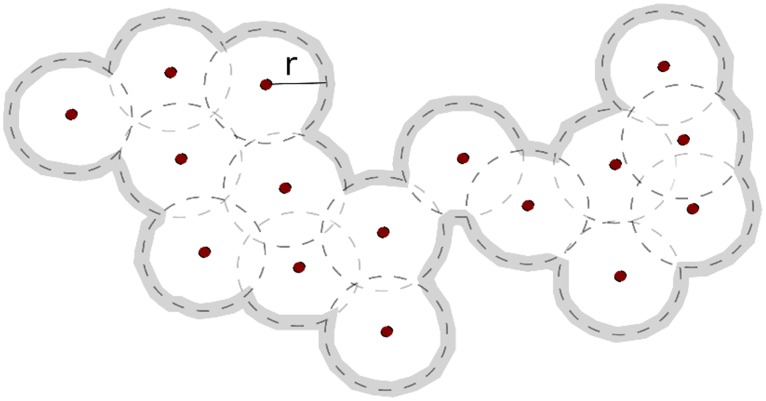
An example for demonstrating inference of travel scope from knowledge of social network geography. Dots represent geographical locations of social ties while *r* denoting the geo-social radius and the inferred travel scope is enclosed by highlighted contour line.

So far, our results suggest that knowledge of social network geography is indicative of travel scope and we can quantify this relationship further. Geo-social radius is an indicator for travel scope for a given geography of social network – as demonstrated by the example shown in Fig. 10 where a travel scope can be determined as an enclosed area spanned by a geo-social radius. The geo-social radius of *y*% coverage of travel scope equals *x* km (i.e. 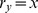 km) means that *y*% of places visited by the subject (or travel scope) are within *x* km from the subjectÕs social ties. We therefore examined the relationship between geo-social radius with area population density for 80% (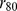) and 90% (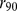) coverage of travel scope.

**Figure 11:**
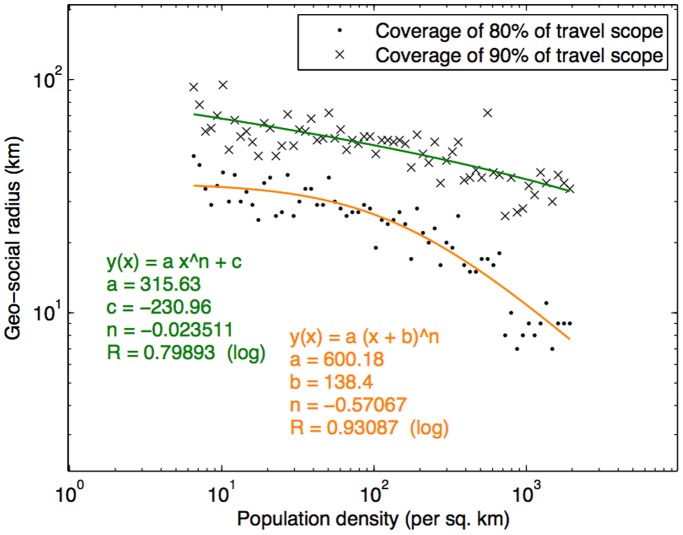
Geo-social radius decreases with population density of the corresponding municipality.

Specifically, for each subject, 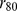 and 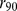 were computed. The subjects were then categorized into group according to the area population density of their home locations. Figure 11 shows the average values of 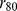 and 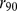 of the subjects across different area population density levels, and the fitted curves. Basically we attempted to examine how the population density level of a residential area influences the geographical distance between the subjects’ travel scope and their social ties, which is reflected in their geo-social radii. We found a strong relationship (see Fig. 11) where the geo-social radius decreases with population density, which also follows the power law. For urban areas such as Lisbon and Porto, 80% of the travel scope can be realized with only about 7****km of geo-social radius (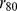), while less densely populated cities such as Parades, Santa Maria da Feira, and Vila Nova de Famalicão would need 15–20****km, and Albufeira, Barcelo and Oliveira do Bairro would need 20–25****km. This suggests that area population density is a key indicator for geo-social radius. Hence *for a given social network’s geography, the travel scope can be inferred in terms of geo-social radius, which can be determined according to the area population density.*

Applying this result to other cities around the world yields the following estimates for 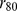 (based on the city’s population density): New York City (2.86****km), Los Angeles (5.08****km), London (4.11****km), Paris (1.99****km), Mumbai (1.99****km), Bangkok (4.25****km), Tokyo (2.41****km), Shanghai (3.51****km), Seoul (2.18****km), Sydney (5.79****km), and Johannesburg (5.51****km).

## Discussion

The fact that mobile phones have become an indispensable part of many people’s lives means that they provide an opportunity to measure human behavior and social dynamics. Call logs and location traces allow researchers to undertake large-scale objective studies of social phenomena. In this study, we used one year’s data for over one million mobile phone users in Portugal to investigate the relationship between human mobility and social networks. We defined *travel scope* as the set of distinct locations visited and found that 80% of people’s travel scope are within just 20****km of their nearest social ties, and that based on their travel scope people are 15% more likely to be physically closer to their weak ties than strong ties. Furthermore, we defined *geo-social radius* as the geographical distance from social ties and found that area population density is a key indicator for the geo-social radius where denser areas imply shorter geo-social radii. Moreover, we found that the likelihood of travel scope being within some geo-social radius increases with area population density where shorter geo-social radii increase at a faster rate. Our results suggest that social network geography along with area population density are indicative of individual travel scope and vice versa.

Nonetheless, there are a number of significant limitations to our study. The first of these is the discontinuous nature of the location traces in our dataset. Since individuals are only located when connections with the cellular network are established, we can only identify a subset of all the locations visited in the course of a day. However, our aggregation of mobility patterns and the longitudinal nature of our data compensates for this. The second limitation is the coarse spatial resolution of the location traces, which is determined by the granularity of cellular tower coverage. Although traditional surveys can achieve much higher spatial resolution, in practice such surveys are only conducted for small samples of the whole population and for very limited periods of time.

Another potential limitation is the effect of population migration (i.e., people moving home from one location to another). However, according to the Portugal Demographic Statistics of 2007 [34], the net migration rate in 2007 was only 0.18% which suggests that it is unlikely to impact on our results significantly. In fact, our previous study of social dynamics during migration [17] identified a 0.03% migration rate in this dataset. There is also the potential impact of people taking vacations, in particular, vacations in the Atlantic archipelagos of the Azores and Madeira, which are about 600–1000****km away from the mainland. This could impact on our result slightly. We did not exclude the subjects who made/received calls from Azores and Madeira from our study because we did not want to risk discarding subjects who were not actually on vacations, and we believed that the vacation periods were just a fraction of the regular life periods, which are unlikely to impact on our results significantly.

A final limitation relates to the extent to which our findings are applicable beyond Portugal. As a First World (developed) country and a member of the Schengen area, Portugal has significant similarities with many European and other developed countries in the world. We thus believe that the findings are likely to be applicable to countries with broadly similar social, cultural, and economic profiles.

This study sheds new light on the socio-geography of human mobility and we hope that our findings suggest new ways to use mobile phone data to investigate the interplay between people’s mobility and their social networks. There are a number of open questions, such as how geo-social radius varies with tie strength and number of ties, how most visited places play a role in social network geography, and the nature of quantitative relationship between mobility and social networks.

### Note

This study was waived of ethical approval from the IRB of Newcastle University.The data was anonymized by the operator before being transferred to the authors, in such way that no connection could be made with any individual. The mobile phone users agreed to the terms and conditions for using the services with the network operator that their communications may be recorded and analyzed for improving services.

## Supporting Information

Figure S1 **Percentage of travel scope being within some distance from ties’ locations based on a null model in which subjects’ locations were randomly interchanged.**
(TIFF)

Figure S2 **Z-scores of the randomized null model (Fig. S1) compared against the result of the real scenario (** **Fig. 7** **).**
(TIFF)

Figure S3 **Neighborhood ratio of weak and strong ties.**
(TIFF)

Figure S4 **Percentage of travel scope being within some distance from weakest and strongest ties’ locations.**
(TIFF)

Figure S5 **Standard errors of the results shown in** **Fig. 7** **(percentage of travel scope being within some distance from ties’ locations, where distance varies from 0 to 200 km).**
(TIFF)

Figure S6 **Standard errors of the results shown in** **Fig. 8** **(percentage of travel scope being within some distance from weak and strong ties’ locations).**
(TIFF)

Figure S7 **Standard errors of the results shown in Fig. S1 (percentage of travel scope being within some distance from ties’ locations based on a null model in which subjects’ locations were randomly interchanged).**
(TIFF)

Figure S8 **Standard errors of the results shown in Fig. S2 (neighborhood ratio of weak and strong ties).**
(TIFF)

Figure S9 **Standard errors of the results shown in Fig. S3 (percentage of travel scope being within some distance from weakest and strongest ties’ locations).**
(TIFF)

Figure S10 **Distance-dependent p-values of the results shown in** **Fig. 8** **.**
(TIFF)
