# Supplementary figures and images for: Socio-Geography of Human Mobility: A Study Using Longitudinal Mobile Phone Data
Source: PLoS One. 2012 Jun 28;7(6):e39253. doi: 10.1371/journal.pone.0039253 (PMC3386290; doi:10.1371/journal.pone.0039253)

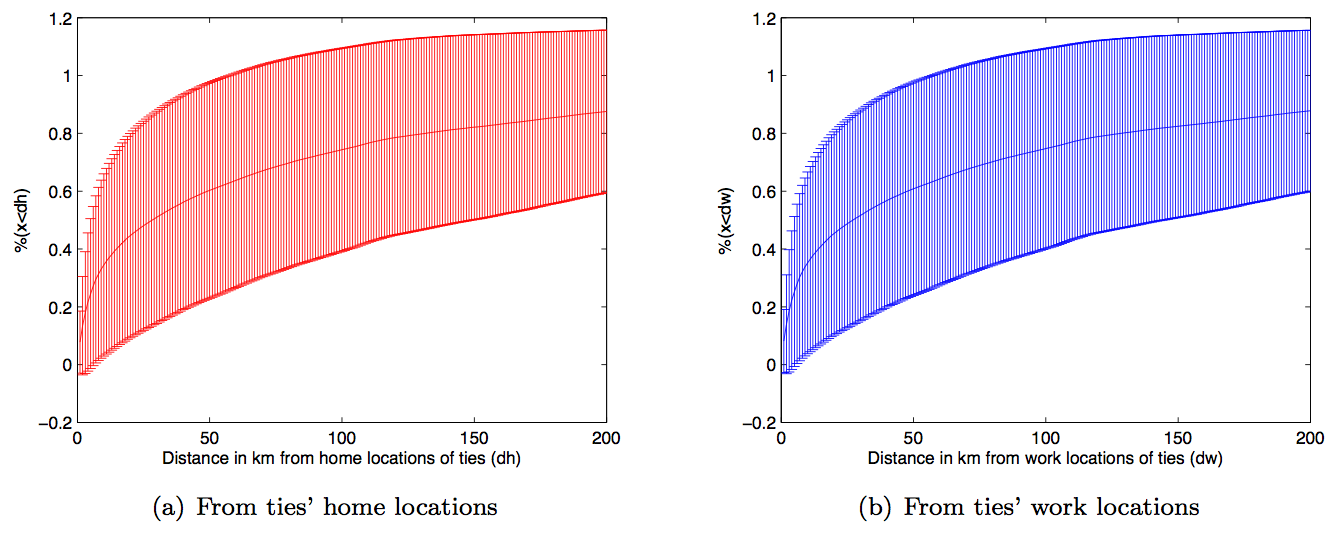

Supplement: Figure S1 — Percentage of travel scope being within some distance from ties’ locations based on a null model in which subjects’ locations were randomly interchanged. (TIFF) [file pone.0039253.s001.tiff]

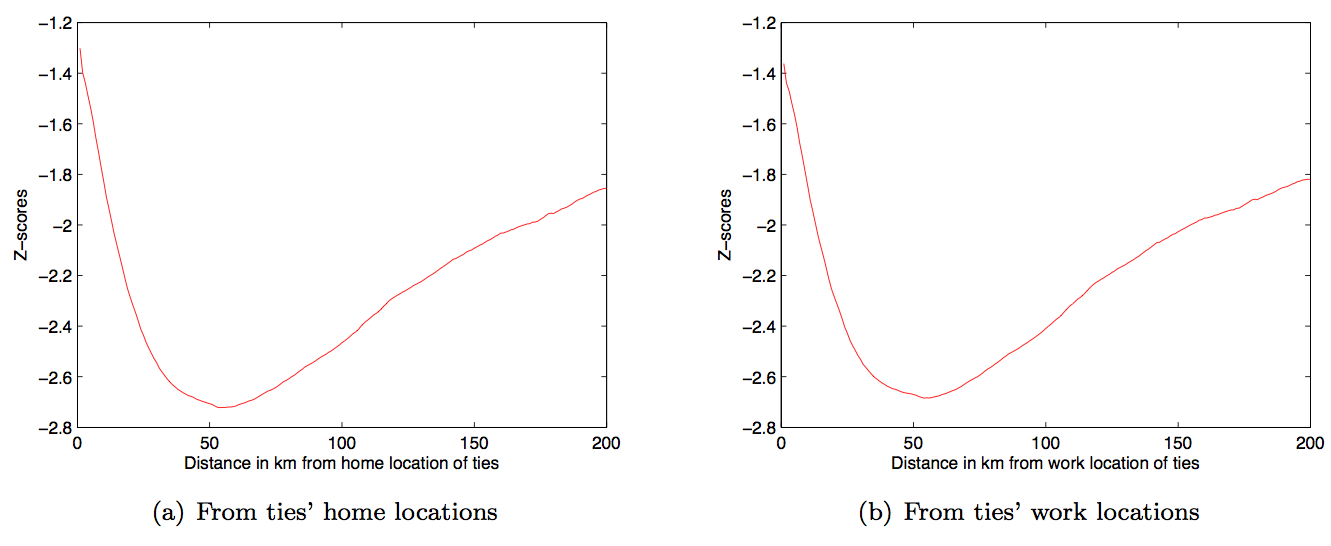

Supplement: Figure S2 — Z-scores of the randomized null model (Fig. S1) compared against the result of the real scenario ( Fig. 7 ). (TIFF) [file pone.0039253.s002.tiff]

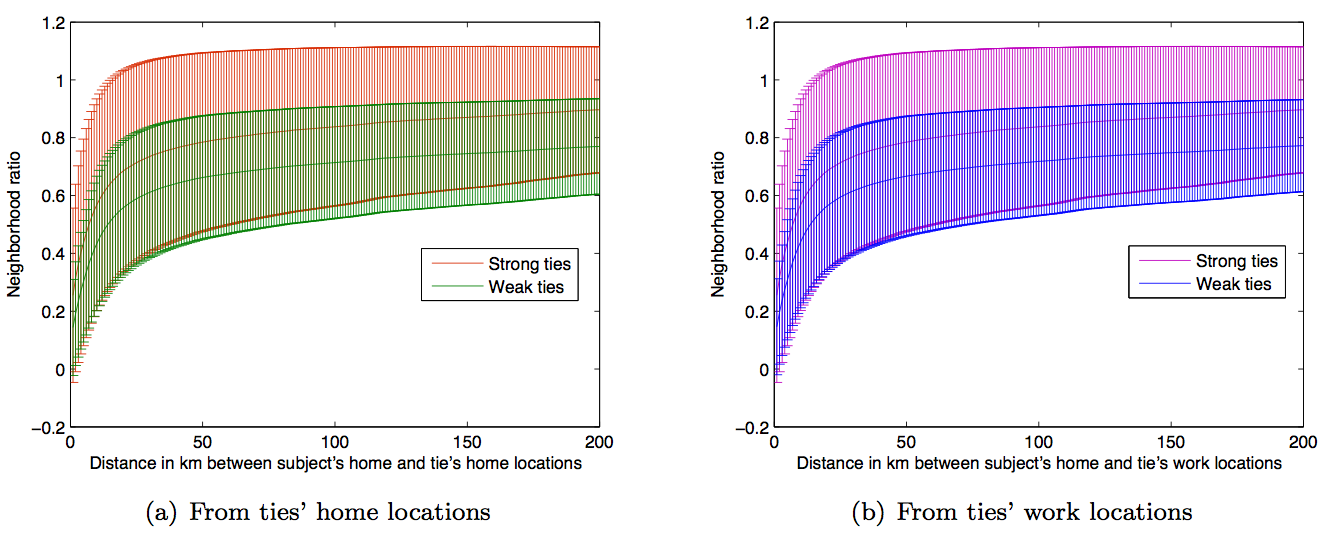

Supplement: Figure S3 — Neighborhood ratio of weak and strong ties. (TIFF) [file pone.0039253.s003.tiff]

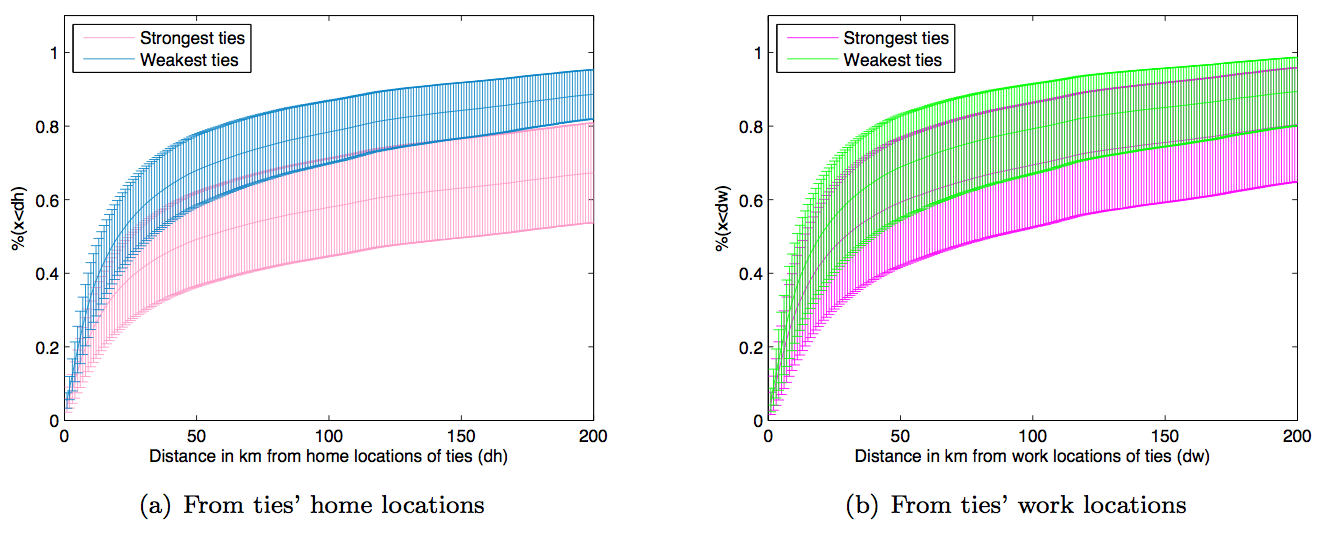

Supplement: Figure S4 — Percentage of travel scope being within some distance from weakest and strongest ties’ locations. (TIFF) [file pone.0039253.s004.tiff]

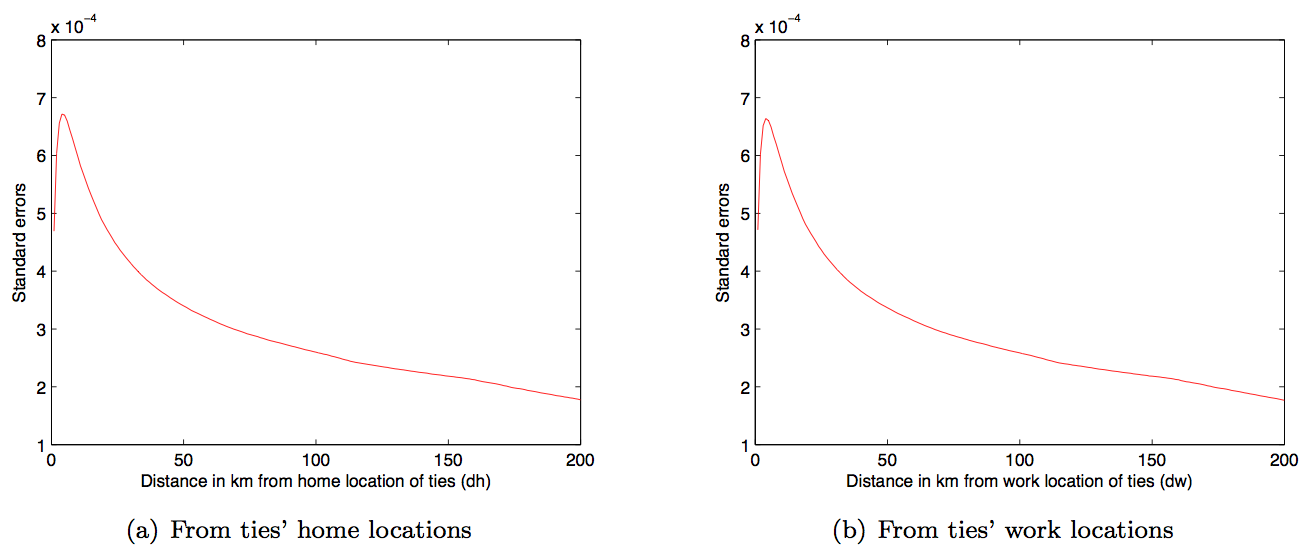

Supplement: Figure S5 — Standard errors of the results shown in Fig. 7 (percentage of travel scope being within some distance from ties’ locations, where distance varies from 0 to 200 km). (TIFF) [file pone.0039253.s005.tiff]

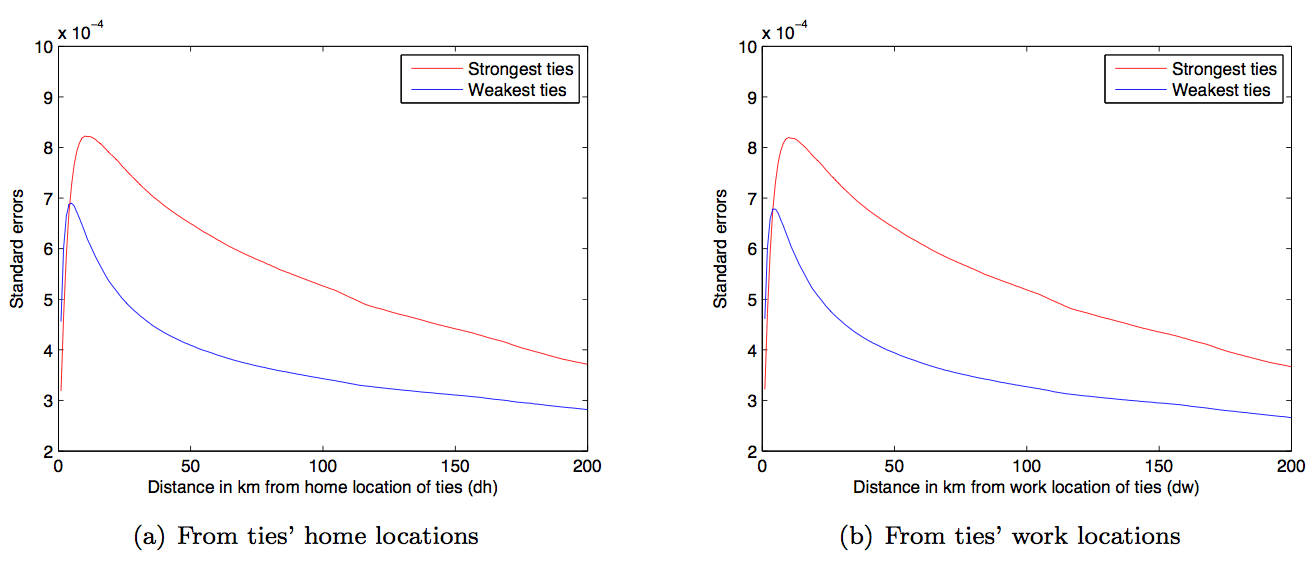

Supplement: Figure S6 — Standard errors of the results shown in Fig. 8 (percentage of travel scope being within some distance from weak and strong ties’ locations). (TIFF) [file pone.0039253.s006.tiff]

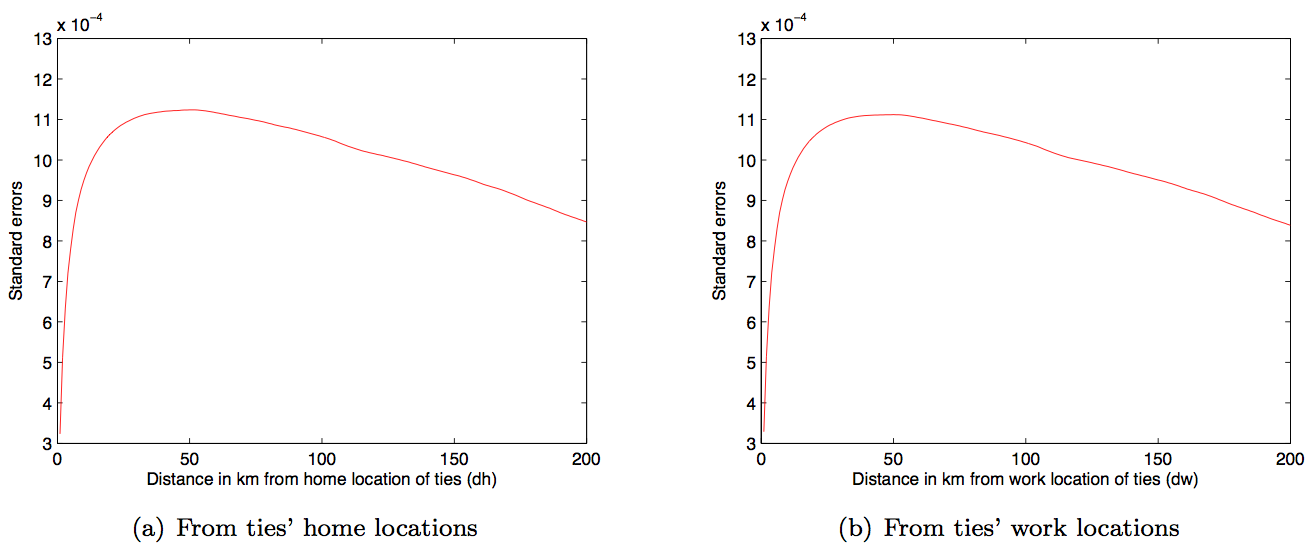

Supplement: Figure S7 — Standard errors of the results shown in Fig. S1 (percentage of travel scope being within some distance from ties’ locations based on a null model in which subjects’ locations were randomly interchanged). (TIFF) [file pone.0039253.s007.tiff]

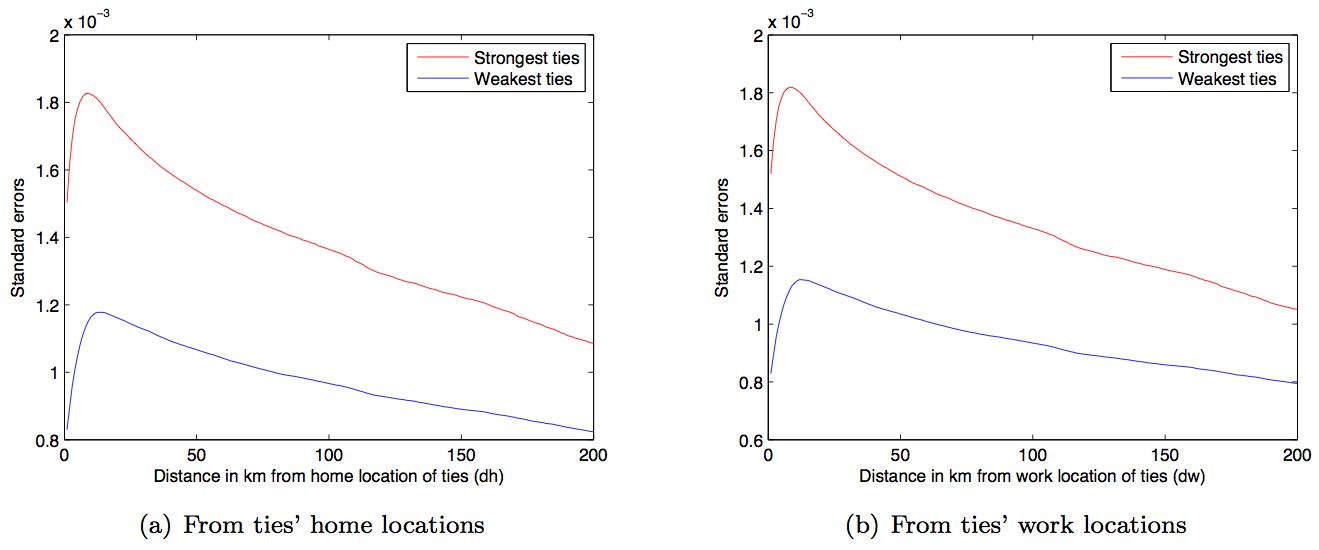

Supplement: Figure S8 — Standard errors of the results shown in Fig. S2 (neighborhood ratio of weak and strong ties). (TIFF) [file pone.0039253.s008.tiff]

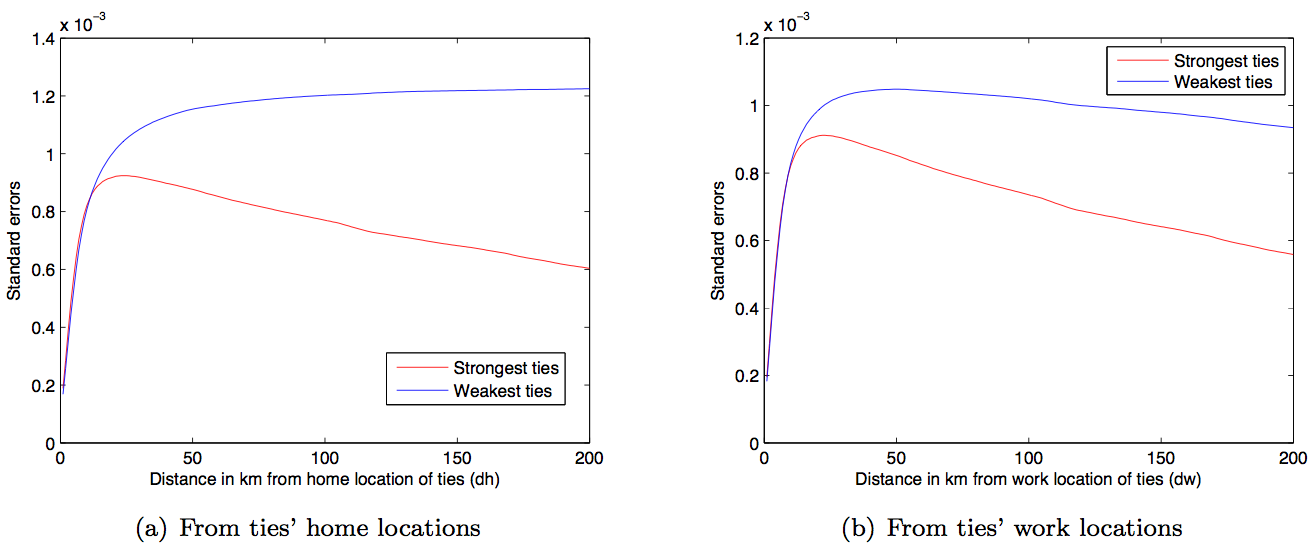

Supplement: Figure S9 — Standard errors of the results shown in Fig. S3 (percentage of travel scope being within some distance from weakest and strongest ties’ locations). (TIFF) [file pone.0039253.s009.tiff]

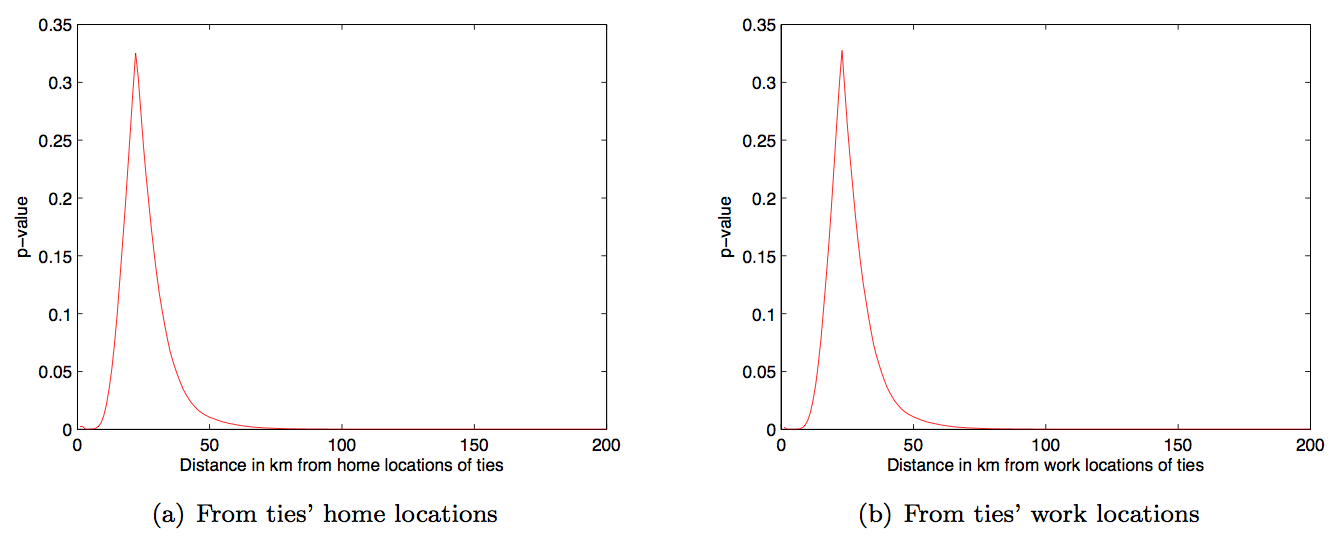

Supplement: Figure S10 — Distance-dependent p-values of the results shown in Fig. 8 . (TIFF) [file pone.0039253.s010.tiff]
